# Early Adversity and Its Impact on Cardiovascular Health: Laying the Groundwork for Dementia Research

**DOI:** 10.1002/alz.089307

**Published:** 2025-01-09

**Authors:** Liang Wang, Sarah Bauermeister

**Affiliations:** ^1^ University of Oxford, OXFORD, Oxfordshire United Kingdom; ^2^ Dementias Platform UK, OXFORD, Oxfordshire United Kingdom; ^3^ University of Oxford, Oxford United Kingdom; ^4^ Dementias Platform UK ‐ University of Oxford, Oxford United Kingdom

## Abstract

**Background:**

Cardiovascular disease (CVD) and dementia share common risk factors like hypertension, diabetes, obesity, smoking, alcohol consumption, physical inactivity, and depression, indicating a complex interplay in their etiologies. This systematic review investigates the connections between early adversity (EA), such as low socioeconomic status (SES) and emotional abuse, and the development of CVD, focusing on how early‐life experiences contribute to CVD comorbidity, a crucial factor in the development of dementia.

**Method:**

Following the PRISMA guidelines, we conducted a comprehensive literature search in databases including Medline, Embase, PsycINFO and Global Health, covering publications from 2000 to November 2023. The search focused on keywords relating to EA, CVD, and aging. Inclusion and exclusion criteria were applied, alongside the use of the QUIPS risk evaluation tool, to ensure rigorous analysis. The primary focus was on studies that explored long‐term cardiovascular outcomes in individuals exposed to EA.

**Result:**

From a comprehensive analysis of 22 articles, we revealed a significant association between EA and critical CVD risk factors like abdominal obesity, smoking, and late‐life hypertension, which are potential causes of dementia. Early life factors, such as lower education levels and socioeconomic status, were found to significantly influence the risk of CVD and associated factors. These early life challenges affect not only physical health, leading to conditions like hypertension and diabetes, but also lifestyle choices related to smoking and alcohol use. Moreover, factors like reduced physical activity, depressive symptoms, and environmental elements such as air pollution were identified as contributing to the development of CVD risks. Collectively, these factors form pathways through which EA increases the likelihood of CVD and subsequent dementia.

**Conclusion:**

This systematic review emphasizes the profound and enduring impact of early life adversities on key risk factors for cardiovascular disease. These insights highlight the need for early interventions and long‐term monitoring of individuals with adverse childhood experiences to reduce future CVD risk. Importantly, this research lays a pivotal foundation for future exploration into dementia risk factors, suggesting an important interrelationship between early adversity, cardiovascular disease and dementia.

